# Unraveling the Potential of miRNAs from CSCs as an Emerging Clinical Tool for Breast Cancer Diagnosis and Prognosis

**DOI:** 10.3390/ijms242116010

**Published:** 2023-11-06

**Authors:** Raquel Nogueras Pérez, Noelia Heredia-Nicolás, Laura de Lara-Peña, Julia López de Andrés, Juan Antonio Marchal, Gema Jiménez, Carmen Griñán-Lisón

**Affiliations:** 1 Biopathology and Regenerative Medicine Institute (IBIMER), Centre for Biomedical Research (CIBM), University of Granada, 18016 Granada, Spain; raquelnog99@gmail.com (R.N.P.); noeliahn1995@gmail.com (N.H.-N.); lauradelara@ugr.es (L.d.L.-P.); e.julialdeandres@go.ugr.es (J.L.d.A.); jmarchal@go.ugr.es (J.A.M.); 2 Biosanitary Research Institute of Granada (ibs. GRANADA), University Hospitals of Granada, University of Granada, 18012 Granada, Spain; 3 Excellence Research Unit “Modeling Nature” (MNat), University of Granada, 18016 Granada, Spain; 4 Department of Human Anatomy and Embryology, Faculty of Medicine, University of Granada, 18016 Granada, Spain; 5 Department of Biochemistry and Molecular Biology II, Faculty of Pharmacy, University of Granada, 18071 Granada, Spain

**Keywords:** breast cancer stem cells, miRNAs, diagnostic, prognostic, signature

## Abstract

Breast cancer (BC) is the most diagnosed cancer in women and the second most common cancer globally. Significant advances in BC research have led to improved early detection and effective therapies. One of the key challenges in BC is the presence of BC stem cells (BCSCs). This small subpopulation within the tumor possesses unique characteristics, including tumor-initiating capabilities, contributes to treatment resistance, and plays a role in cancer recurrence and metastasis. In recent years, microRNAs (miRNAs) have emerged as potential regulators of BCSCs, which can modulate gene expression and influence cellular processes like BCSCs’ self-renewal, differentiation, and tumor-promoting pathways. Understanding the miRNA signatures of BCSCs holds great promise for improving BC diagnosis and prognosis. By targeting BCSCs and their associated miRNAs, researchers aim to develop more effective and personalized treatment strategies that may offer better outcomes for BC patients, minimizing tumor recurrence and metastasis. In conclusion, the investigation of miRNAs as regulators of BCSCs opens new directions for advancing BC research through the use of bioinformatics and the development of innovative therapeutic approaches. This review summarizes the most recent and innovative studies and clinical trials on the role of BCSCs miRNAs as potential tools for early diagnosis, prognosis, and resistance.

## 1. Introduction

**Figure 1:**
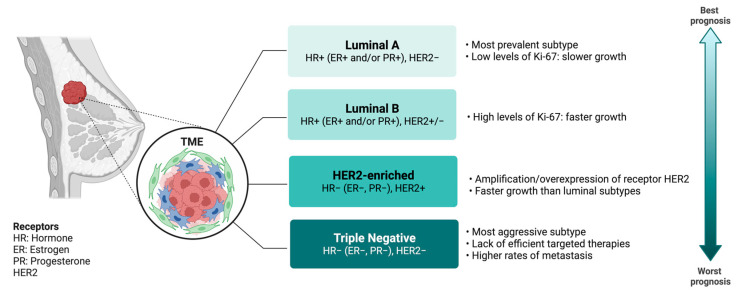
BC molecular subtype classification.

Breast cancer (BC) is the most common cancer in women, with 2.26 million cases in 2020, and the second most common worldwide. The number of deaths in developed countries has significantly decreased in recent years, which can be attributed to both early detection and the development of new effective therapies and targets [1]. BC is classified into four different types based on the histological and biochemical characteristics of the tumor cells (Figure 1). The least aggressive type is luminal BC, which can be further divided into two subtypes. Luminal A BC is characterized by being estrogen receptor (ER)- and/or progesterone receptor (PR)-positive, HER2-negative, and having low levels of expression of the Ki-67 protein, which is associated with cell proliferation. This slower growth leads to a better prognosis for patients. On the other hand, luminal B BC is ER- and/or PR-positive, HER2-positive or -negative, and has high levels of Ki-67 expression. This type of tumor grows faster than luminal A, resulting in a slightly worse prognosis for patients [2,3]. Another type of BC is HER2-positive, characterized by being ER- and/or PR-negative and HER2-positive. This leads to faster tumor growth compared to luminal BC, resulting in a poorer prognosis. However, there are effective treatments targeting the HER2 protein [2,4,5]. Finally, the worst prognosis occurs in triple-negative BC (TNBC), which is negative for hormonal receptors (both ER and PR) and HER2. The characteristics of this type of BC include a lack of efficient targeted therapies and higher rates of metastasis compared to other types. Additionally, recurrences often occur shortly after considering the patient as cured [2,6,7].

After diagnosis, a variety of therapies can be used, including chemotherapy, radiotherapy, or surgery [1,8]. However, in many cases, patients experience a recurrence years after tumor regression, largely due to tumor heterogeneity. In the tumor, the population of differentiated tumor cells responsible for tumor growth coexists with a minority population of cancer stem cells (CSCs) characterized by their self-renewal capacity, tumorigenicity, differentiation into other tumor cell types, and resistance to conventional therapies. CSCs are implicated in tumor recurrence after the completion of treatment and metastasis [9,10,11]. In the case of BC, the population of CSCs can be distinguished from the population of differentiated tumor cells through the expression of specific surface markers for breast CSCs (BCSCs), like CD44high, CD133, or CD61, as well as the absence of other specific membrane markers such as CD24-/low, CD64, or CD18. Additionally, BCSCs are also characterized by high levels of aldehyde dehydrogenase 1 (ALDH1) expression [12].

The importance of early diagnosis of the disease, as well as establishing an accurate prognosis for the patient to guide treatment with the most effective and personalized therapy for their type of BC, has led to the search for new biomarkers over the years. To date, the most commonly used biomarkers in BC have been the presence/absence and number of metastases in the axillary lymph nodes, tumor size, and tumor stage, which have significant limitations and lack reproducibility [13,14]. Other biomarkers that have been used are multi-gene tests, which analyze a panel of genes that may be mutated and associated with the development and progression of the disease. However, these tests are expensive and cannot be performed on all patients or in all countries [2,15]. In addition, obtaining tumor samples for the analysis of these markers requires invasive techniques, which further limits their use [2,16]. For all these reasons, current research is focused on the search and analysis of new diagnostic and prognostic biomarkers for BC, as well as markers that can evaluate tumor development and metastatic capacity, and differentiate between differentiated tumor cell populations and BCSCs [16].

In this context, microRNAs (miRNAs) have emerged as a potential tool due to their stability in tissues and blood plasma, allowing for less invasive techniques such as liquid biopsy to be used for their retrieval [17]. These small RNA molecules (21–23 nucleotides) do not encode proteins but instead play a regulatory role in genes at the post-transcriptional level, affecting the final expression of messenger RNA (mRNA). They are involved in the regulation of multiple cellular processes, such as cell differentiation, proliferation, apoptosis, and tissue development, and have also been implicated in pathological processes such as carcinogenesis [18]. Therefore, any change in miRNA expression will modify the amount of the final product encoded by mRNAs, consequently disrupting homeostasis and normal physiological cellular processes [17,18,19]. Once these miRNAs have been identified, it is necessary to use techniques that can accurately quantify their expression. Currently, the most widely used technique is quantitative real-time polymerase chain reaction (qPCR) due to its high sensitivity and specificity, which guarantees the obtained reproducibility of the results provided that sample manipulation errors are minimized. When analyzing results obtained through this technique and to avoid misinterpretations, it is important to consider the inherent endogenous variations in each individual and ensure that the chosen endogenous controls for result normalization are appropriate for each specific case [17].

Due to the importance of BCSCs in the carcinogenic process, as well as early BC detection, and the limited research on the dysregulation of miRNA expression and BCSCs, this review summarizes the most recent and innovative studies on the role of miRNAs as potential tools for early diagnosis, prognosis, and resistance of patients to conventional treatments, as well as their oncogenic and metastatic contributions. Furthermore, clinical trials evaluating the utility of miRNAs as potential biomarkers in clinical practice are presented, along with new bioinformatics tools to assist researchers in the search for newly dysregulated miRNAs related to BCSCs.

## 2. miRNA Expression Related to BC Stem-like Phenotype

miRNAs play a crucial role in the development, invasion, and maintenance of BCSCs by acting directly on signaling pathways that regulate cellular homeostasis. These pathways are tightly regulated in normal stem cells, but deregulated in BCSCs, resulting in aberrant activation or inhibition. The Wnt/β-catenin, JAK/STAT, Notch, transforming growth factor-β (TGF-β), PI3K/Akt, and nuclear factor kappa-light-chain-enhancer of activated B cells (NF-*κ*B) signaling pathways are the main pathways involved in the maintenance of BCSCs characteristics and stemness [20]. Likewise, these pathways can also lead to the overexpression or downregulation of different miRNAs whose targets affect the acquisition of the stem-like phenotype or tumor malignancy. This section focuses on the compilation of miRNAs that have been identified as indicators of the BCSC phenotype and their relationship to the signaling pathways mentioned above. Table 1 summarizes the miRNAs related to the regulation of these signaling pathways, either because they are involved in the pathways or because their regulation depends on them.

### 2.1. Wnt/β-Catenin

**Figure 2:**
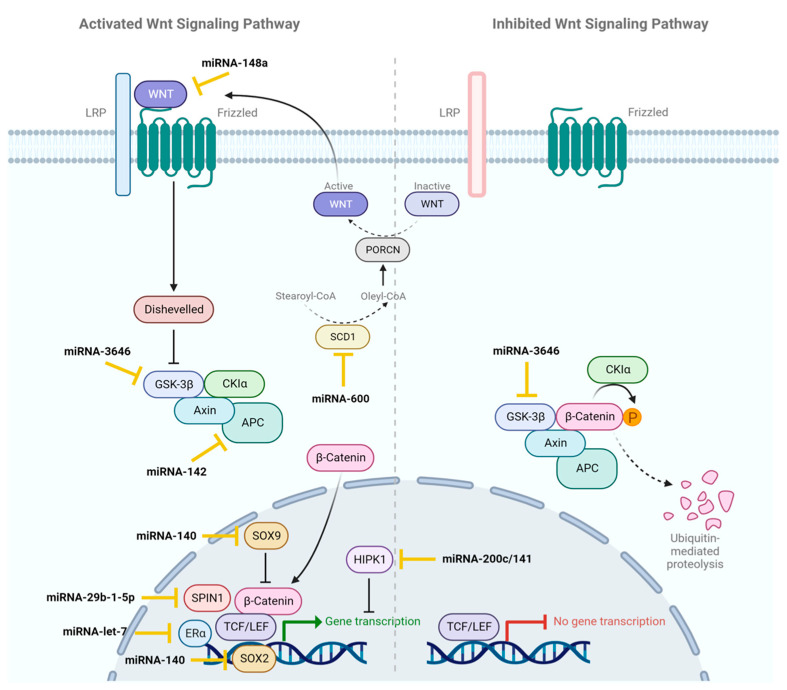
Wnt/β-catenin signaling pathway and miRNAs that are involved.

The Wnt/β-catenin signaling pathway (Figure 2) is involved in embryonic development and the maintenance of cellular homeostasis. Aberrant overexpression of this pathway causes CSC renewal, proliferation, and differentiation. In this pathway, Wnt glycoproteins are secreted by cells into the extracellular matrix and bind to G protein-coupled receptors (GPCRs), leading to their activation. This inhibits the destruction of β-catenin, which accumulates in the cell nucleus and interacts with transcription factors, thus activating the expression of genes involved in cell proliferation and differentiation [20,54].

One of the main enzymes involved in this pathway is glycogen synthase kinase 3β (GSK-3β). This enzyme is part of the protein complex that, in the absence of Wnt, phosphorylates β-catenin, leading to its ubiquitination and subsequent destruction. The inhibition of GSK-3β results in the accumulation of β-catenin in the cytoplasm and its translocation to the nucleus, consequently activating the Wnt/β-catenin pathway and CSC proliferation. Zhang et al. found a negative correlation between miRNA-3646 and the expression of the enzyme GSK-3β. When cells were transfected with miRNA-3646 mimics, the expression of the GSK-3β gene was significantly reduced, leading to an increased proliferation of BCSCs compared to the control condition. Conversely, the transfection of cells with miRNA-3646 inhibitors increased the GSK-3β levels and cellular apoptosis. Therefore, this study associates miRNA-3646 with chemotherapy resistance in BC cells, a common feature of BCSCs [24].

Another study, conducted by Jiang et al., highlights the importance of the proto-oncogene WNT-1, which is overexpressed in tumoral tissues compared to adjacent normal tissues. WNT-1 is involved in the activation of the Wnt/β-catenin pathway and is associated with phenomena related to BCSC’s proliferation and migration. In this case, the analyzed miRNA was miRNA-148a, which negatively correlates with the expression of WNT-1 and, therefore, with the activation of the Wnt/β-catenin pathway in BC tissues. To evaluate the relationship between miRNA-148a expression and the WNT-1 gene, Jiang and colleagues analyzed miRNA-148a expression levels by in situ hybridization and WNT-1 expression by immunohistochemistry in BC patient samples. The results showed that WNT-1 expression increased when miRNA-148a expression decreased and, conversely, WNT-1 expression decreased when miRNA-148a expression increased. This suggests that this miRNA would act by downregulating the expression of the WNT-1 gene and thereby inactivating the Wnt/β-catenin pathway, resulting in the inhibition of BCSC proliferation [22].

El Helou et al. described miRNA-600, which acts as a switch that can increase or decrease the quantity of BCSCs. The study identifies SCD1 as a target of miRNA-600, which plays a key role in regulating the Wnt/β-catenin signaling pathway as its overexpression reduces BCSCs’ expansion. This study suggests that altering miRNA-600 and its target could have therapeutic applications in BC, as SCD1 may be relevant to CSCs’ self-renewal. It also emphasizes the importance of controlling WNT signaling in BCSCs and proposes other therapeutic approaches, such as inhibiting the protein PORCN. In summary, this study provides valuable insights into the regulation of BCSCs and presents potential treatment strategies [21].

On the other hand, miRNA-140 acts as a tumor suppressor by inhibiting CSCs signaling and cancer initiation through its interaction with SOX2 and SOX9 in both ductal carcinoma in situ (DCIS) and invasive ductal carcinoma (IDC). When miRNA-140 decreases, CSCs increase, leading to cancer growth. Two main causes of decreased miRNA-140 levels are estrogen binding in IDC and CpG island methylation in DCIS. If these mechanisms are disrupted, miRNA-140 levels are restored, reducing CSCs and tumor growth. This could be crucial in preventing progression to IDC. Epigenetic therapy rescuing miRNA-140 is an innovative strategy for both DCIS and IDC, especially in tamoxifen-insensitive cases without ERα [23].

The results of the study of Isobe et al. indicate that miRNA-142 plays a significant role in regulating the canonical Wnt/β-catenin signaling pathway and proliferation in mammary cells, both normal and malignant. Although miRNA-142 primarily focuses on suppressing the APC protein, a key component of the Wnt/β-catenin signaling pathway, other pathways are likely involved due to miRNAs’ ability to have multiple targets. This study suggests that the upregulation of miRNA-142 could be an important feature of BCSCs and directly contribute to their abnormal proliferation. These findings provide valuable insights into the underlying molecular mechanisms in BC and the significance of Wnt/β-catenin signaling pathway regulation in BCSCs [25].

Another miRNA related to the Wnt/β-catenin signaling pathway is miRNA-29b-1-5p. It was found that TNBC has low levels of miRNA-29b-1-5p, which could contribute to its development, as miRNA-29b-1-5p expression is inversely correlated with mammosphere stemness potential. TNBC cells with lower miRNA-29b-1-5p were more likely to regenerate and less sensitive to treatments. Increasing miRNA-29b-1-5p levels in these cells reduced their growth and mobility, making them more responsive to certain treatments. In addition, a gene called SPIN1 has been identified as a possible target of miRNA-29b-1-5p [27].

In a study by Liu et al., two distinctive populations of BCSCs with characteristics similar to mesenchymal or epithelial cells were identified, each with different properties in terms of tumor growth and invasion. The study investigated how these populations could mutually transform in epithelial–mesenchymal transition (EMT), involving the miRNA-200c/141 and the HIPK1/β-catenin axis. It was observed that inhibiting miRNA-200c/141 increased the invasion of BC cells but reduced their ability to proliferate. It was identified that miRNA-200c/141 affects genes related to EMT, and its inhibition leads to the activation of the Wnt/β-catenin signaling pathway. Overall, this study suggests that the regulation of miRNA-200c/141 plays an important role in BCSC heterogeneity and BC progression [29].

Ultimately, a study by Sun et al. found that let-7b and let-7c are associated with a better prognosis in BC and that let-7c is inversely related to Erα expression, which plays a role in BC. It is suggested that let-7c might influence a type of BCSC that is positive for ERα. The study proposes that let-7c regulates these cells’ ability to renew themselves by affecting the Wnt/β-catenin pathway. The experiments showed that let-7c can inhibit the renewal of BCSCs by degrading the mRNA of ERα, leading to a reduction in the activation of the Wnt/β-catenin signaling pathway and the formation of BC tumors [30].

### 2.2. JAK/STAT

**Figure 3:**
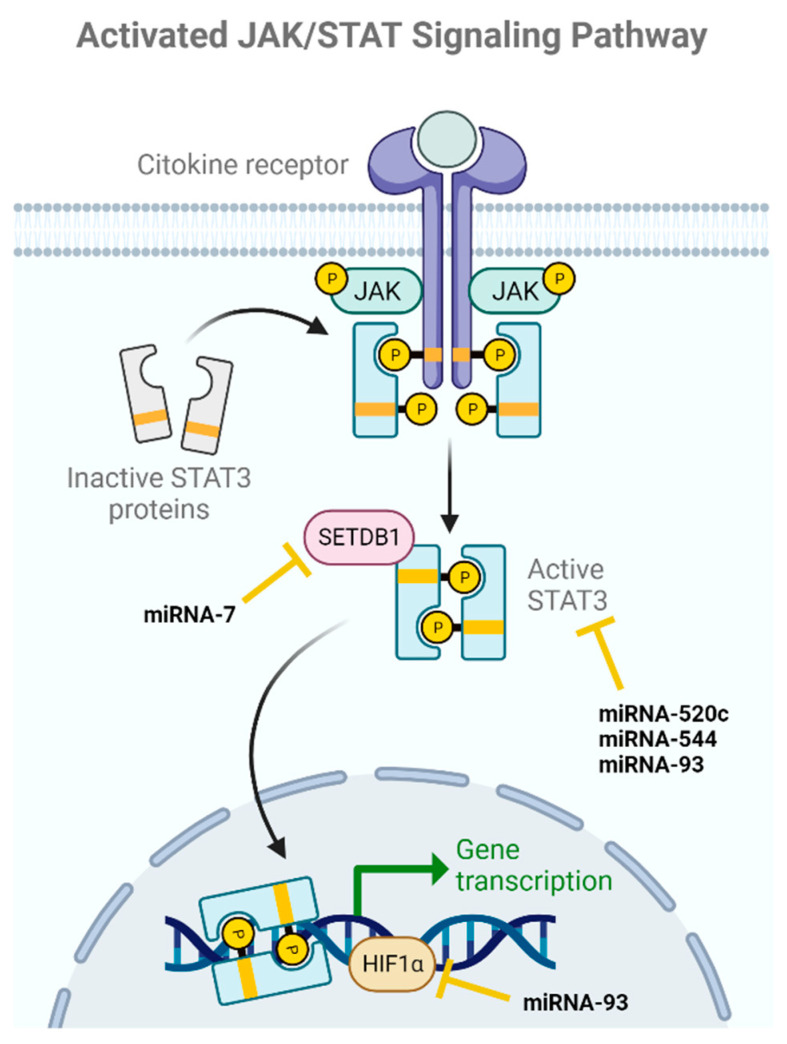
JAK/STAT signaling pathway and miRNAs that are involved.

Another pathway involved in the regulation of BCSCs characteristics is the JAK/STAT pathway (Figure 3), which involves JAK tyrosine kinases that bind to the cytoplasmic regions of type I and II cytokine receptors. Once bound, the receptors dimerize and recruit JAKs, which undergo phosphorylation and recruit one or more STATs for phosphorylation. These STATs then translocate to the nucleus, where they activate or repress the transcription of target genes. Dysregulation of this pathway leads to the acquisition of pluripotency in BCSCs [20,55].

The relationship between BC and the Signal Transducer and Activator of Transcription 3 (STAT3) has been extensively studied, as the aberrant activation of STAT3 is associated with BC formation and progression [56]. In this line, Wang and colleagues investigated miRNA-520c as a potential regulator of STAT3. They examined the expression levels of miRNA520c and STAT3 and observed that lower levels of miRNA-520c were associated with higher tumor malignancy and increased invasiveness into adjacent tissues. The overexpression of miRNA-520c significantly reduced the levels of STAT3 protein and the mesenchymal marker vimentin, while increasing the epithelial marker E-cadherin. Taken together, these results suggest that this miRNA suppresses the EMT process and the acquisition of pluripotency in BCSCs by directly affecting the JAK/STAT pathway through its interaction with STAT3 [33].

In another study, Zhu et al. investigated the role of miRNA-544 in STAT3 in patients with TNBC. They found that the overexpression of STAT3 in these samples was associated with an increase in the expression of Bcl6, a protein that plays a crucial role in EMT, tumor progression, decreased survival, and the viability of BCSCs. Additionally, a decrease in the expression levels of miRNA-544 was observed in the tumor tissues. The results showed that increasing the expression of miRNA-544 led to a decrease in the expression of both STAT3 and Bcl6, resulting in a significant reduction in the proliferation, migration, and invasion of cancer cells in TNBC. miRNA-544 expression could reverse EMT, decreasing the expression of a stem phenotype in tumor cells. These findings suggest that miRNA-544 acts as a tumor suppressor in TNBC by negatively regulating the expression of STAT3 and Bcl6 [34].

Liu et al.’s study focused on how the miRNA-93 affects BCSCs. They discovered that miRNA-93 influences the proliferation and differentiation of these cells. In different types of BC cells, the levels of miRNA-93 varied depending on their degree of differentiation. It was demonstrated that the continued expression of miRNA-93 simultaneously downregulates a number of BCSCs’ self-renewal pathways, including JAK/STAT, promoting cellular differentiation and depleting the BCSC population. Furthermore, miRNA-93 had different effects depending on the cell type [35].

The backbone of Liu et al.’s work was miRNA-106a and its role in BC. They explored how the interaction between fibroblasts and BC cells influences the formation of BCSCs. miRNA-106a play a pivotal role in this process, affecting critical pathways and genes like STAT3 and HIF-1α [36].

Lastly, miRNA-7, which is downregulated in BCSCs, has been found to act as a metastasis inhibitor and target EMT in BCSCs. Researchers observed that the overexpression of miRNA-7 reduces SETDB1 activity and partially reverses EMT in cancer cells [37].

### 2.3. Notch

**Figure 4:**
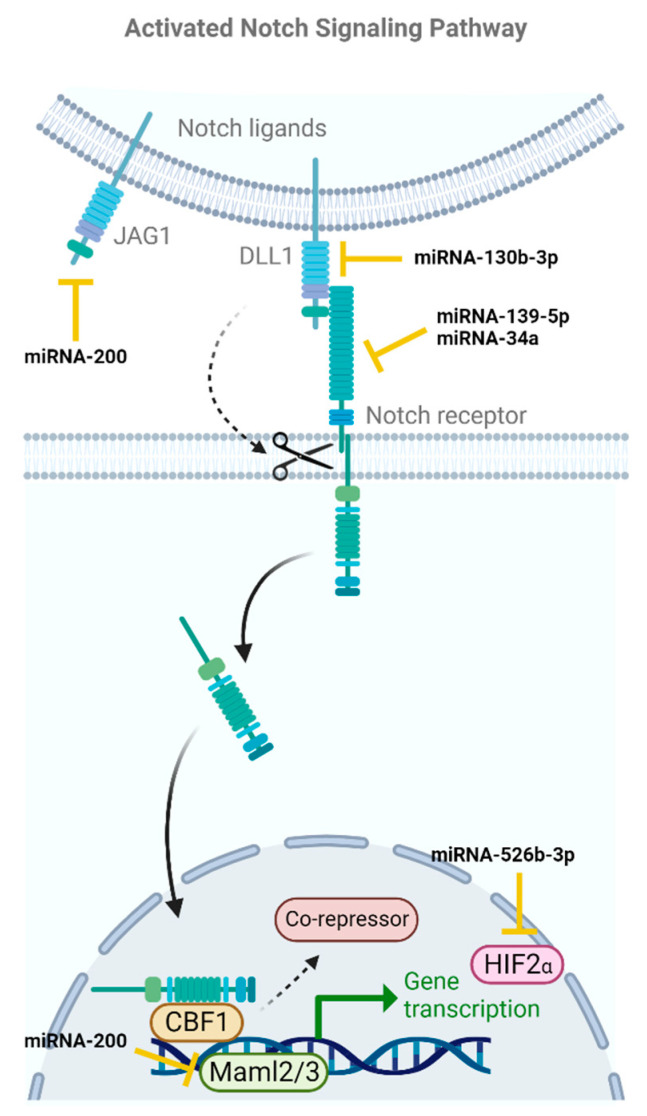
Notch signaling pathway and miRNAs that are involved.

The third pathway involved in maintaining the characteristics of BCSCs is the Notch signaling pathway (Figure 4), which is activated through cell-to-cell contact. In this pathway, ligands, such as DLL1, DLL3 and Jagged1, bind to the extracellular domain of Notch receptors, causing the separation of the intracellular domain of the receptor and its translocation to the cell nucleus. Once in the nucleus, the intracellular domain of the Notch receptor interacts with the co-repressors of the transcription factor CBF1, displacing them and binding to CBF1, thereby enabling the activation of the Notch signaling pathway. This pathway is crucial for cell proliferation, differentiation, and apoptosis, so the overexpression of this pathway leads to tumor progression in BC and the differentiation of BCSCs [20,57].

Through various experimental analyses, Shui et al. discovered that DLL1, a key ligand of the Notch signaling pathway, is a target of miRNA-130b-3p in BC cells. The results of this study showed that as the expression of miRNA-130b-3p increased, both cellular invasion and migration significantly decreased due to the negative regulation of DLL1 by miRNA-130b-3p. On the other hand, when the expression of this miRNA decreased, an increase in all processes related to tumor metastasis was observed. These findings demonstrated that miRNA-130b-3p plays a role in inhibiting the migration and invasion of BC cells by regulating the expression of the Notch ligand DLL1 in BC [39].

Thanks to the work of Zhang et al., it was found that the expression of miRNA-139-5p is dysregulated in BC compared to adjacent healthy tissues, and this dysregulation is correlated with the maintenance of CSCs’ pluripotency, angiogenesis, and tumor non-encapsulation. The researchers performed various experiments to study cell proliferation, and they found that when cells were treated with miRNA-139-5p mimics, not only was cell proliferation inhibited, but apoptosis was induced, and tumor cells were sensitized to chemotherapeutic drugs. These experiments also revealed an increase in the number of cells in the S phase, confirming that miRNA-139-5p expression can stop tumor growth. Furthermore, it inhibits the expression of the Notch1 membrane receptor, which is involved in the Notch signaling pathway [40].

Another miRNA related to the Notch pathway is miRNA-34a, which is involved in cell-cycle arrest, cellular self-renewal, apoptosis, and EMT. miRNA-34a is downregulated in BCSCs due to mutations in the p53 gene, leading to increased cancer stem cell properties, tumor development, and metastasis. Park et al. observed that this occurs because miRNA-34a targets Notch1, so a decrease in its expression leads to the overexpression of CD44, thereby accelerating tumor development [38].

Thanks to the work carried out by Liu et al., it has been identified that miRNA-526b-3p plays a crucial role in regulating resistance to conventional chemotherapy treatments, as well as in regulating the properties of BCSCs. In this study, it was demonstrated that the expression levels of miRNA-526b-3p decrease, correlating with an increase in Notch pathway expression, by inducing the upregulation of HIF-2α, which is involved in the progression of BCSCs and tumor hypoxia, resulting in drug resistance. Through in vitro studies, researchers have observed that the increased expression of this miRNA in BC leads to an attenuation of BCSC growth, reduced cell proliferation, and the inhibition of resistance to conventional drugs [41].

Finally, miRNA-200 family suppresses Notch pathway signaling by directly targeting its components, including JAG1 and the Notch coactivators Maml2 and Maml3. The downregulation of miRNA-200 members leads to a reduction in the metastasis and migration of BCSCs in TNBC [42].

### 2.4. PI3K-AKT

**Figure 5:**
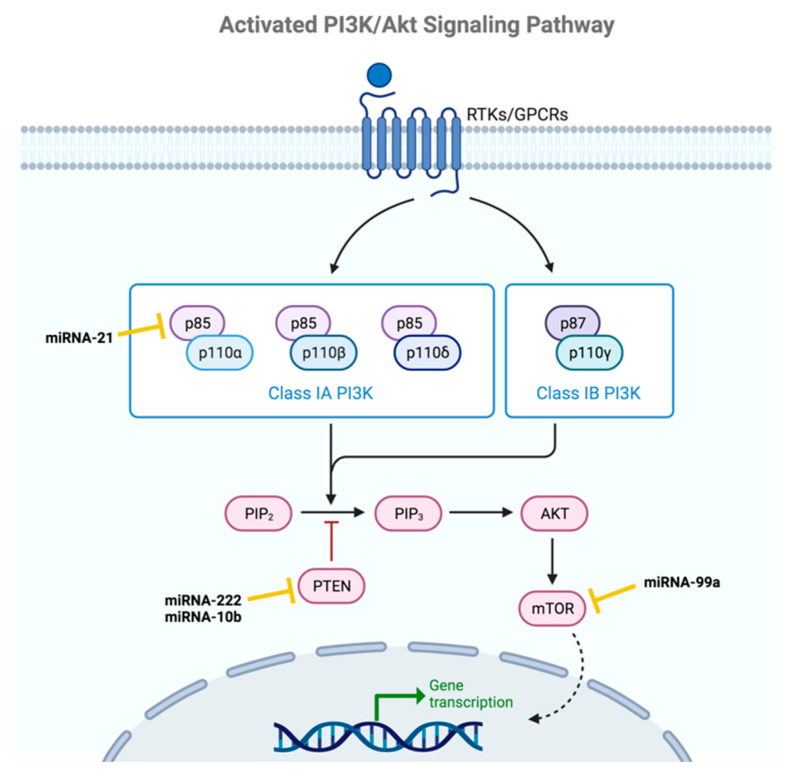
PI3K/Akt signaling pathway and miRNAs that are involved.

The PI3K-AKT signaling pathway (Figure 5) is also involved in the regulation and maintenance of BCSCs characteristics. This pathway involves a large number of enzymes that are highly regulated in normal cells, so any alteration in the regulation of the function of these enzymes can lead to processes associated with tumor development, as well as the behavior of BCSCs. Firstly, the phosphoinositide 3-kinase enzymes family (PI3Ks), which controls cell division, are activated by the binding of specific ligands to their corresponding receptor (RTK or GPCR). Activated PI3Ks phosphorylate phosphatidylinositol 3,4-bisphosphate (PIP2) to form phosphatidylinositol 3,4,5-trisphosphate (PIP3), which leads to the binding and activation by phosphorylation of protein kinase B (PKB) or AKT, which is involved in cell survival. The activation of AKT modulates numerous factors responsible for the acquisition of CSC characteristics. AKT activation can be reversed by numerous proteins, including PTEN, which acts as a scaffold protein in the cell nucleus, controlling genomic stability, apoptosis, and cell-cycle progression [20,58,59].

miRNA-221/222 is one of the most studied clusters in relation to BCSCs. Although numerous studies had already reported the role of this cluster in the acquisition of the stem phenotype, Li et al. described the underlying molecular mechanism. In their study, they demonstrated that the target of miRNA-221/222 is PTEN and that overexpression of this miRNA enhanced the growth, migration, and invasion of BC cells by down-regulating PTEN. In addition, Shen et al. discovered that, in HER2-positive BC, miRNA-222 is overexpressed, leading to resistance to chemotherapeutic drugs used to treat this type of cancer. In their work, they observed that when the expression levels of miRNA-222 decreased, the tumor cells became more sensitive to the drugs, while increasing its levels above normal led to resistance to the therapies used. They attributed this to the regulation of the PI3K/AKT signaling pathway by PTEN through miRNA-222. Overexpression of this miRNA leads to decreased expression of the tumor suppressor gene PTEN, leading to increased phosphorylation of the AKT enzyme and, consequently, overactivation of the PI3K/AKT pathway. Additionally, these researchers also investigated how inhibiting AKT phosphorylation increased the sensitivity of tumor cells to drugs and reduced the proliferative, migratory, and invasive activity of BCSCs. The results obtained in this study suggest that PTEN is a target of miRNA-222, which inhibits its expression and activates AKT phosphorylation, thereby enhancing the pro-tumor effects of this pathway [43,44].

Another oncomiRNA related to the overexpression of this pathway is miRNA-21, studied by Yan et al. The overexpression of miRNA-21 leads to a decrease in the expression of the p85α protein, which is necessary for the stabilization and recruitment of the p110α subunit of the PI3K protein, preventing its activity. Consequently, the loss of the p110α subunit activates the PI3K/AKT signaling pathway. These researchers have observed that in 7.8% of BC tumors, the expression of the p85α protein is reduced, which they associated with the overactivation of the PI3K/AKT pathway due to the loss of the inhibitory effect of p85α on p110α. Furthermore, in other analyses, they found that the p85α protein acts as a positive regulator of PTEN by stabilizing it, supporting the idea that miRNA-21 activates the PI3K pathway through multiple targets. In vitro assays conducted by these researchers have shown that reducing miRNA-21 led to an increase in p85α expression levels, along with a decrease in phosphorylated AKT levels (the active form of the AKT protein) and a reduction in PI3K/AKT pathway activation. All these results highlight that miRNA-21 is involved in tumor growth, as well as the migration, and invasion of BCSCs, by activating the enzymes involved in the PI3K/AKT pathway [45,46].

miRNA-10b is an oncogene whose overexpression in BC leads to the overactivation of the PI3K/Akt pathway. Bahena-Ocampo et al. observed that the overexpression of miRNA-10b results in inhibition of PTEN expression, which is involved in metastasis and cell survival. Therefore, overexpression of this miRNA promoted the BCSCs phenotype and increased their self-renewal by maintaining the activation of the AKT pathway [47].

On the other hand, it has been observed that the decrease in miRNA-99a expression is associated with the maintenance of stem cell-like characteristics in BCSCs. miRNA-99a plays a fundamental role in cell differentiation and may be involved in EMT. The decrease in this miRNA expression level leads to an increase in mTOR activity, which is a key effector in the PI3K/Akt signaling pathway, resulting in the maintenance of the CSC phenotype in BC [48].

### 2.5. TGF-β

**Figure 6:**
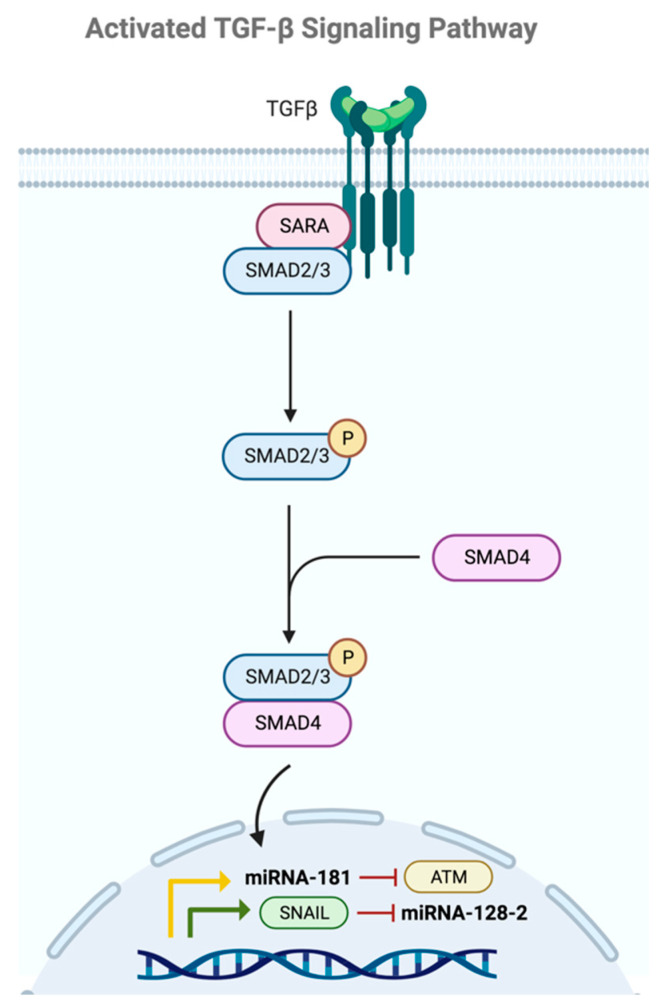
TGF-β signaling pathway and miRNAs that are involved.

The transforming growth factor-beta (TGF-β) signaling pathway (Figure 6) is another fundamental pathway involved in the regulation of numerous cellular functions, including growth, differentiation, and apoptosis. When this pathway is dysregulated, it leads to tumorigenic processes associated with CSCs. This signaling pathway is activated when the TGF-β ligand binds to its receptor on the cell surface, triggering receptor activation and phosphorylation of the Smad protein family. The phosphorylation of this protein family leads to the formation of protein complexes that translocate to the cell nucleus, where they act as transcription factors regulating the expression of genes related to the growth and differentiation of CSCs [49].

The regulation of CSCs by TGF-β depends on intrinsic signaling pathways within cancer cells, including the ATM signaling axis. The study conducted by Wang et al. reveals that the overexpression of miRNA-181 induced sphere formation in BC cells, and that TGF-β was responsible for such upregulation. However, the effect of miRNA-181 on BCSCs appears to be independent of Smad4, a component that is commonly associated with the regulation of miRNA-181 in other cellular contexts. Alternatively, a new target of miRNA-181 was identified, ATM, a protein involved in the response to DNA damage. The suppression of ATM by miRNA-181 may contribute to the formation of BCSCs and potentially to chemotherapy resistance. However, it is noted that the regulation of ATM in BCSCs can be complex and have opposing effects on the response to cancer therapy [49].

In the study conducted by Qian et al., miRNA-128-2 was identified as a tumor suppressor that becomes deregulated in BC. They propose that TGF-β might be involved in EMT and tumorogenesis by downregulating miRNA-128 expression through phosphorylation of the TGF-β1 receptor to increase SNAIL expression. SNAIL downregulates miRNA-128-2, which, in turn, represses different stem cell factors. The proposed SNAIL/miRNA-128 axis provides novel insights into the mechanism of mammary epithelial oncogenic transformation. Deregulation of this miRNA leads to increased proliferation, treatment resistance, metastasis, and the invasion of BCSCs [50].

### 2.6. NF-κβ

**Figure 7:**
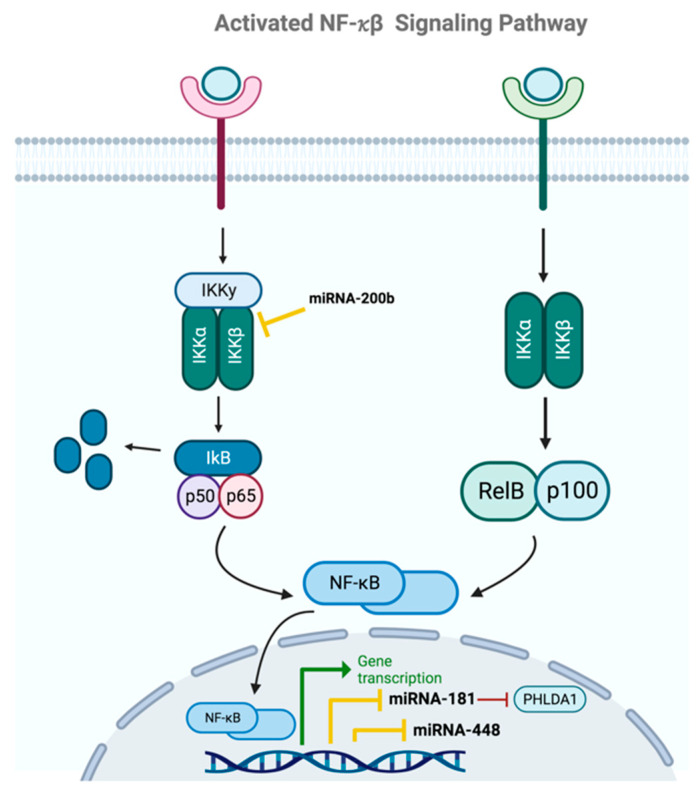
NF-*κ*β signaling pathway and miRNAs that are involved.

In this pathway involved in the maintenance of BCSCs in the tumor (Figure 7), NF-*κ*β transcription factors are inactive in the cytoplasm until they are activated by the arrival of specific ligands to the cell, which bind to their receptors and activate the I*κ*β kinase (IKK), phosphorylating the I*κ*β enzyme. Once this enzyme is activated, NF-*κ*β transcription factors form homodimers or heterodimers and translocate to the cell nucleus, where they activate target genes. The aberrant activation of this cellular pathway is associated with various cellular processes related to cell survival and proliferation, as well as the acquisition of stem-like phenotypes, allowing for the maintenance of active BCSCs within the tumor mass [20,60,61].

Several miRNAs are involved in the regulation of this signaling pathway, including miRNA-181, which was studied by Kastrati et al. These researchers proved that PHLDA1 is a direct target of miRNA-181 and that this miRNA is downregulated in an ER- and NF *κ*β-dependent manner. Thus, ER and NF-*κ*β work together synergistically to upregulate PHLDA1 through both an increase in its transcription and the repression of miRNA-181. Furthermore, they determined that PHLDA1 is upregulated in mammospheres of ER+ BCCs and is associated with an increased risk of distant metastasis in patients with ER+ BC. Their study concludes that elevated PHLDA1 expression is controlled through an ER-NF-*κ*β-miRNA-181 regulatory axis, which increases the stem-like BC phenotype, and thus alters the prognosis of BC patients [51].

Other studies have focused on investigating the role of miRNAs as inhibitors of IKK expression, such as the study conducted by Wu et al., in which they analyzed miRNA-200b. This miRNA inhibits the activation of the NF-*κ*β pathway by directly targeting IKK-β, resulting in the inhibition of cellular colony formation in BCSCs, as well as their migration. Consistent with previous studies, these researchers observed that miRNA-200b expression is downregulated in TNBC, particularly in migratory cells. Wu and colleagues demonstrated that miRNA-200b suppresses IKK-β expression by binding directly to the 3’-UTR region of the mRNA encoding this kinase, leading to the inhibition of I*κ*β enzyme phosphorylation and, consequently, the translocation of NF-*κ*β transcription factors to the nucleus. All these results led to the conclusion that miRNA-200b can modulate the expression of the NF-*κ*β pathway, such that optimal expression of this miRNA decreases the expression of the NF-*κ*β pathway, whereas downregulation of miRNA expression leads to aberrant NF-*κ*β signaling. Therefore, miRNA-200b may be a potential therapeutic tool for treating patients with TNBC and inhibit tumorigenesis in this cancer [52].

In Mak et al.’s study, the primary focus was on miRNA-448 and its role in the microenvironment’s influence on BCSC generation. They demonstrated that the presence of M2 tumor-associated macrophages (TAMs) enriched the CSC subpopulation in two human BC cell lines. This enrichment led to a significant increase in the migratory and invasive abilities of BC cells, primarily driven by the NF-*κ*β pathway. Significantly, the compound pterostilbene was shown to counteract these effects, mediated by M2 TAMs, by reducing NF-*κ*β expression, and pterostilbene-mediated NF-*κ*β downregulation was correlated with an increased amount of miRNA-448. Mak et al. propose that pterostilbene holds promising potential as an anti-CSC agent, addressing not only the primary cancer cells but also the tumor microenvironment (TME), with particular emphasis on its impact on M2 TAMs [53].

## 3. miRNAs as Diagnostic Biomarkers in BCSCs 

The diagnosis of BC in its earliest stages is of great importance, as it allows halts the progression of the disease and prevents complications such as metastasis or resistance to conventional treatments. Currently, imaging diagnostic tests such as mammography and biochemical tests such as hormone receptor measurement are the most commonly used in clinical practice [62,63,64]. However, these tests are not very sensitive or specific, and they are invasive and therefore unpleasant and uncomfortable for the patients who undergo them [64]. These reasons have led many scientists to search for new diagnostic biomarkers that allow for the early diagnosis of BC. In this line, the study of miRNAs as diagnostic biomarkers is emerging [64,65]. It has been observed that these nucleic acids can be detected in plasma, serum, and other body fluids, as well as in tumor tissue [66,67]. miRNAs have properties that make them good diagnostic biomarkers, as they remain stable even under severe conditions such as pH variations or multiple freeze–thaw cycles, and they are found in different body fluids, either free, bound to proteins, or encapsulated in exosomes, which facilitates their extraction and analysis, allowing for their retrieval through less invasive and less costly techniques such as liquid biopsy. Moreover, their expression can significantly increase or decrease during oncogenesis in BC [62,67].

By mapping the signature and profile of circulating miRNAs, it has been found that some of them are differentially expressed in patients with BC compared to healthy individuals (Table 2). This is the case of miRNA-182, studied by Wang and colleagues, who were able to isolate this miRNA from both the tumor tissue and serum of patients and observed that, in both cases, miRNA-182 was significantly overexpressed compared to healthy controls [68]. Previous studies have shown that miRNA-182 acts as an oncogene that promotes the development of BCSCs and BC when overexpressed [69,70], whereby β-catenin binds to the promoter of this miRNA and increases its expression, leading to oncogenic processes in the breast tissue. Additionally, elevated levels of miRNA-182 decrease the expression of the BRCA1 gene, thereby hindering DNA repair [71]. These studies led Wang and colleagues to analyze the potential of this miRNA as a possible diagnostic biomarker for BC, and, after isolating it, the results showed that miRNA-182 levels were significantly higher in patients compared to healthy controls, with this difference being more significant when the miRNA was isolated from serum rather than tumor tissue. They also found a correlation between miRNA-182 levels and hormone receptors, both ER and PR, such that when the expression of these receptors was positive, miRNA serum levels were lower compared to patients who were negative for both receptors. With all these data, this study highlighted the role of miRNA-182 as a potential diagnostic biomarker for BC [68].

Another study that highlights the importance of using circulating miRNAs as a tool for the early diagnosis of BC was conducted by Swellam et al., who analyzed the role of miRNA-27a in the diagnosis of BC and its oncogenic process. These researchers found that miRNA-27a expression varies significantly, being higher in BC patients, followed by patients with benign breast tumors, and reaching the lowest levels in healthy individuals [72]. This phenomenon is attributed to the fact that miRNA-27a overexpression promotes the viability of tumor cells by overactivation of the cell cycle and reducing apoptosis, since it directly targets the FOXO1 gene, which is involved in the regulation of proliferation and survival of BCSCs, and reduces its expression, as described in previous studies [73]. The analysis conducted by Swellam and colleagues demonstrated the positive relationship between miRNA-27a and both ER and HER2. Additionally, the diagnostic efficacy of miRNA-27a was studied in comparison with the routine BC tumor markers currently used in clinics (CEA and CA15.3), showing that miRNA-27a has higher specificity and accuracy for diagnosing BC in its early stages, even when the disease is undetectable by other techniques, and is also useful for identifying patients at a high risk of developing BC. Therefore, in addition to being a good diagnostic marker, it can also be used as a predictive biomarker [72].

The oncogenic role of miRNA-373 has also been studied several times in different types of cancer [89,90,91]. However, the function of this miRNA in BC was questionable, which led Bakr et al. to investigate it further. These researchers observed a significant increase in miRNA-373 expression levels in patients with primary BC and identified a positive correlation between its expression levels and metastasis, which is related to the inhibition of CD44 BCSCs’ surface marker expression and activation of the Wnt/β-catenin signaling pathway involved in tumorigenesis, mediated by miRNA-373 [74]. Furthermore, another study demonstrated that the increased expression of the miRNA correlated with the overexpression of the VEGF gene in breast tumor tissue, which plays a key role in angiogenesis, and thus tumor growth and metastasis, highlighting the fact that miRNA-373 acts as an essential factor in tumorigenesis and metastasis and can potentially be used as a diagnostic biomarker for BC [75].

In addition to the study of miRNAs as individual diagnostic biomarkers, studies are also being conducted to group different miRNAs into panels, which may allow for the development of a more sensitive and specific diagnostic tool compared to the study of a single miRNA in isolation. In this line, the work of researchers Abbas et al. stands out, who developed a panel consisting of miRNA-29b and miRNA-31 for the early diagnosis of BC. In this study, overexpression of miRNA-29b was observed in BC patients compared to healthy controls, which correlated with nodal involvement, tumor size, disease stage, and metastasis [76]. Previous studies suggest that the overexpression of miRNA-29b leads to a decrease in PTEN gene expression, thereby activating the PI3K pathway and promoting the proliferation and migration of BCSCs [77]. On the other hand, the researchers found that miRNA-31 was also overexpressed in BC patients compared to controls and promoted BCSCs division and tumorigenesis by stimulating the Wnt signaling pathway [26,76,78]. The results obtained in this study lead to the conclusion that both of the studied miRNAs are overexpressed in BC and related to BCSCs, and the combined measurement of both can be used as a highly specific diagnostic marker for BC. Furthermore, the dysregulation of miRNA-29b and miRNA-31 are risk factors indicating a high likelihood of disease recurrence [76].

Another panel of great interest is formed by miRNA-21 and miRNA-155, which can be considered the most significantly altered in BC. These miRNAs were studied by Soleimanpour et al., who found that the levels of both miRNAs were significantly higher in both plasma and tumor tissue compared to healthy controls [59]. As previous studies have shown, miRNA-21 functions as a key oncomiRNA that is overexpressed in a large number of tumors, including BC. This miRNA stimulates BCSCs’ proliferation, cell growth, and apoptosis [79,80,81]. On the other hand, miRNA-155 regulates BC progression, cell growth, survival, cell adhesion, and drug resistance. Additionally, miRNA-155 overexpression is positively correlated with HER2-positive BC, such that high levels of this miRNA in plasma are associated with the diagnosis of ER+ and/or PR + BC [82]. In the analysis performed by Soleimanpour and colleagues, it was found that both miRNA-21 and miRNA-155 were significantly overexpressed in BC patients, both in plasma and tumor tissue, suggesting that both circulating miRNAs have great potential to discriminate between BC patients and healthy individuals. Therefore, they could be used in the clinic as non-invasive biomarkers for BC. Additionally, this panel could also be used as a diagnostic biomarker in women with a family history of BC, providing a preventive and very early diagnostic tool [62].

Han and colleagues studied the same panel described earlier in the work conducted by Soleimanpour et al., but they added the miRNA-365, which was shown through previous in vitro experiments to reduce the proliferation and migration capacity of BCSCs when overexpressed [64], acting as a tumor suppressor miRNA. Consistent with the work of Soleimanpour et al., Han and colleagues demonstrated that both miRNA-21 and miRNA-155 were overexpressed in both the tumor tissue and plasma of BC. In addition, the novelty of this study lies in the inclusion of miRNA-365 in the panel, which showed decreased levels when analyzed in the plasma and tumor tissue of BC patients compared to healthy controls [64]. By studying the panel formed by these three miRNAs, the researchers observed that the diagnostic sensitivity and specificity for BC increased compared to the biomarkers more commonly used in the clinic, such as CEA and CA 153. Furthermore, both sensitivity and specificity also increased when analyzing the set of the three miRNAs compared to analyzing the possible combinations of pairs of these miRNAs. Therefore, this study provides evidence that the evaluation of miRNA levels in plasma can be used as a diagnostic biomarker for BC and that the combination of multiple miRNAs, in this case, miRNA-21, miRNA-155, and miRNA-365, can increase sensitivity and specificity, allowing for improved differentiation between BC patients and healthy individuals [64].

Recent advances in miRNA research as diagnostic biomarkers for BC are directed towards the search for increasingly larger panels that include a greater number of dysregulated miRNAs and allow for very early differentiation between sick and healthy patients. The study conducted by Itani et al. is an example of this. In this study, the researchers designed a macro-panel that includes five dysregulated miRNAs in BC: miRNA-21, miRNA-155, miRNA-23a, miRNA-425-5p, and miRNA-139-5p. In the case of miRNAs-21, 155, and 23a, as in other previous studies, they are significantly overexpressed in BC patients compared to healthy controls, both in plasma and tumor tissue, suggesting an oncogenic role in BC oncogenesis [66,83,84,85,86]. miRNA-23a promotes BC progression by directly activating the FOXM1 gene, which regulates the cell cycle, BCSCs’ proliferation, and DNA damage response [87]. Similarly to the previous ones, miRNAs 425-5p and 139-5p are overexpressed in the blood plasma of BC patients, with miRNA-425-5p acting as an oncomiRNA by promoting cell invasion and migration through PTEN, whereas the overexpression of miRNA-139-5p inhibits migration and metastasis (BCSCs related process) by disrupting the TGF-β, Wnt, and MAPK/PI3K signaling pathways [66]. The analyses developed by these researchers revealed that the combination of all the studied miRNAs had higher diagnostic accuracy than when studied individually or in smaller separate groups, which, in line with the studies mentioned above, highlights the importance of evaluating dysregulated miRNAs as a whole [66].

## 4. miRNA as Prognostic Biomarkers in BCSCs

Once BC has been diagnosed, the next crucial step is to perform an accurate prognosis of the disease, which will allow for us to understand how the disease will progress, and which therapies are best suited to the specific characteristics of the patient’s tumor [92]. One of the most promising tools currently under study is the use of miRNAs as prognostic biomarkers, as changes in their expression levels are associated with different pathological states of the disease, enabling the detection, among other things, of when the population of BCSCs is higher in the tumor mass. Furthermore, miRNAs can be obtained by non-invasive techniques, and the results obtained show higher sensitivity and specificity compared to traditional biomarkers in vitro [93,94]. Currently, numerous investigations are focused on identifying new miRNAs that can be used as prognostic biomarkers in clinical practice, either as substitutes or complements to commonly used prognostic biomarkers. Some of the most innovative lines of research carried out in recent years, linking the search for new prognostic miRNAs with BCSCs, are presented in the following Table 3 [95].

Farré et al. described a signature of dysregulated miRNAs in breast tumors and their correlation with target genes as potential prognostic biomarkers for BC. They compared the expression of miRNAs 21-5p and 106b-5p between normal breast tissue and tumor tissue in different human samples. In addition to using bioinformatics tools to analyze the pathways in which the studied miRNAs and their target genes were involved, they also examined overall survival and median relapse-free time based on the expression of dysregulated miRNAs and genes, as well as their correlation with the aggressiveness of BC in each patient. Following their respective analyses, these researchers observed that both miRNA-21-5p and miRNA-106b-5p were overexpressed in breast tumor tissue compared to normal tissue. As a result, eight target genes (GAB1, GNG12, HBP1, MEF2A, PAFAH1B1, PPP1R3B, RPS6KA3, and SES N1) were downregulated in BC [96].

Consistent with previous studies, miRNA-21-5p was found to be overexpressed in HER2+ BC, acting as an oncomiRNA, and its dysregulation was associated with proliferation, invasion, and EMT, phenomena associated with the CSCs population [97,98]. The overexpression of this miRNA in HER2 + BC is associated with reduced overall survival and is thought to be responsible for the downregulation of the genes MEF2, PAFAH1B1, PPP1R3B, and RPS6KA3. Furthermore, this miRNA was also overexpressed in more aggressive subtypes of BC, where it downregulated the genes GAB1, GNG12, HBPQ, and SESN1, correlating with decreased overall survival and shorter relapse-free survival [96,99,100,101]. On the other hand, miRNA-106b-5p is more overexpressed in TNBC, associated with downregulation of the genes GAB1, GNG2, HBPQ, and SESN1, shorter overall survival, reduced disease-free time, and progression of BC. Both miRNAs share target genes and act as tumor suppressor genes when overexpressed, resulting in reduced overall survival and disease-free survival, leading to a worse prognosis for the disease [96,102,103,104].

miRNA-7641 exhibits elevated expression in BC, both within the tumor mass and within exosomes released by highly metastatic cancer cells [105]. This study revealed that suppressing miRNA-7641 expression led to a significant reduction in cell proliferation and differentiation, and concurrently enhanced the effectiveness of certain treatments like doxorubicin. Additionally, it was evident that the transfer of miRNA-7641 via exosomes to recipient cells located at various distances caused epigenetic modifications, transforming them into tumor cells and fostering metastasis and tumor growth. The findings of this investigation underscored that patient with metastatic BC demonstrated higher levels of miRNA-7641 in their plasma compared to non-metastatic patients and healthy individuals. Furthermore, it was observed that this miRNA is correlated with reduced patient survival and impacts several crucial cellular processes associated with BCSCs. Therefore, miRNA-7641 holds promise as a potential prognostic biomarker linked to BCSCs [105].

Another miRNA whose role as a prognostic biomarker is subject to considerable debate is miRNA-10b, which has been the subject of numerous in vivo and in vitro studies, with varying results. Some authors have reported that miRNA-10b is overexpressed in metastatic BC [106], while others have found that it is downregulated in tumor tissue compared to normal breast tissue [107]. Therefore, Dwedar et al. investigated the role of miRNA-10b in BC and its targets. In this study, the researchers observed a significant increase in circulating miRNA-10b expression in BC patients compared to healthy controls. They analyzed the relationship between the expression of this miRNA and the clinical characteristics of each patient and concluded that miRNA-10b levels gradually increased with the disease stage, reaching the highest levels in stage IV patients. They also found a positive correlation between axillary lymph node involvement, tumor size, and HER2 positivity [108]. The findings of these researchers are consistent with previous work by Khalighfard et al. [89], who concluded that miRNA-10b is an oncomiRNA whose expression is significantly increased in BC patients, and Zhang et al. [109], who proposed that the expression of this oncomiRNA increases with tumor stage. What distinguishes Dwedar et al. in this study is their analysis of miRNA-10b targets, through which they identified different genes and proteins that are strongly involved in BC metastasis [108]. Among all the analyzed targets, they focused on E-cadherin (E-cad) due to its crucial role in controlling cell adhesion and its established function as a suppressor of invasion and metastasis in BC. The data obtained by these researchers showed that serum levels of E-cad in BC patients were significantly higher compared to control cases and patients with metastasis compared to those without metastasis. When they evaluated the association between serum miRNA-10b and serum E-cad, they found that the overexpression of this miRNA was positively associated with the serum E-cad levels of BC patients and correlated with tumor size, stage, and lymph node metastasis. Based on these findings, Dwedar and colleagues suggest the combined use of serum miRNA-10b and E-cad as a non-invasive prognostic biomarker in BC patients [108].

In the study carried out by Msheik et al., as predicted by other previous work, they observed that miRNA-126 was downregulated in breast tumor tissue compared to adjacent normal tissue [110,111]. Moreover, the plasma levels of this miRNA discriminated between patients with early-stage BC and patients with metastatic BC. These researchers associated the low miRNA-126 expression with lower overall survival [110,112]. In this study, Msheik and colleagues validated miRNA-126 expression using RT-qPCR in samples from ER+ BC patients and evaluated the correlation between miRNA-126 expression and different cellular processes involved in BC and related to BCSCs. In these analyses, they observed a highly significant downregulation of miRNA-126 compared to healthy controls. To better understand its role in ER+ BC, they transfected cells from the commercial MCF-7 cell line in vitro with a miRNA-126 mimic. They observed a significant decrease in cell proliferation and tumor sphere formation, suggesting that increased miRNA-126 expression may act as a tumor suppressor in ER+ BC and reduce the population of BCSCs in the tumor [110]. Furthermore, these researchers also noticed that as miRNA-126 levels increased, the metastatic capacity of the tumor decreased. Therefore, the low expression associated with ER+ BC may be associated with a worse prognosis of the disease [110].

In the last study we refer to, Gong C. et al. developed and validated a novel prognostic model based on 10 specific miRNAs associated with BCSCs. This model aimed to enhance the accuracy of predicting disease recurrence in patients with non-metastatic HR+ HER2- BC. Their retrospective analysis indicated that patients in the low-risk group derived minimal benefit from chemotherapy, while those identified as having a higher recurrence risk based on their classifier seemed to benefit more from chemotherapy. Currently, gene expression profiling assays like the 21-gene test and MammaPrint signature are used in clinical practice to predict recurrence risks in BC patients. However, 10 specific miRNA classifiers appear to outperform these assays in certain scenarios, such as HR+ HER2- patients with negative lymph nodes. This suggested that the miRNA-21, miRNA-30c, miRNA-181a, miRNA-181c, miRNA-125b, miRNA-7, miRNA-200a, miRNA-135b, miRNA-22 and miRNA-200c signature could be valuable in predicting chemotherapy sensitivity, although further prospective studies are needed to confirm this [113].

## 5. miRNA and Chemo-Radioresistance in BCSCs

Once a patient has been diagnosed with BC, chemotherapy and radiotherapy are the most commonly used treatments to reduce the tumor size and destroy as many cancer cells as possible to facilitate subsequent surgery [114,115]. Although there have been numerous advances in diagnosis, early prognosis, and treatment, statistical evidence shows that the five-year survival rate for BC patients remains relatively low [116]. This highlights the need to find new treatments for this disease. This reduced survival rate is largely due to the inability of these therapies to eliminate all tumor cells, leading to increased resistance to conventional treatments, recurrence after the patient is considered cured, and metastasis. Consequently, chemo- and radioresistance stands as major clinical challenge in BC treatment [114,115,117].

In the context of resistance therapies in BCSCs, research is investigating specific miRNAs that are dysregulated and increase the patient’s risk of resistance to certain treatments, as well as others that are associated with patient sensitivity to the same treatments (Table 4).

Recent studies have shown that chemotherapy stimulates tumor cells to release exosomes rich in miRNAs, inducing the conversion of initially treatment-sensitive cells into resistant ones, while also modulating responses to the TME [116]. The study of exosomes about miRNAs and treatment resistance is crucial because exosomes are an important component of the TME, contribute to the acquisition of drug resistance and tumor progression [118], and can sequester cytotoxic drugs, thereby reducing drug concentrations in the tumor [119]. Furthermore, exosomes produced by drug-resistant cells can induce resistance in distant sensitive cells by transporting dysregulated miRNAs [120].

**Table 4:** miRNAs biomarkers of chemotherapy resistance/sensitivity.

MiRNA	MiRNA Acts as a Tumor Suppressor or Oncogenic miRNA	An Increase in Expression Causes	References
miRNA-378a-3p, miRNA-378d	oncogenic	chemoresistance	[114]
miRNA-205	oncogenic	chemoresistance	[121]
miRNA-708-3p	tumor suppressor	chemosensitivity	[117]
miRNA-1207	oncogenic	chemoresistance	[122]
miRNA-137	tumor suppressor	chemosensitivity	[123]
miRNA-142-3p	tumor suppressor	radiosensitivity	[124]
miRNA-29b-3p	oncogenic	radiosensitivity	[125]
miRNA-5088-5p	tumor suppressor	radioresistance	[126]
Let-7	tumor suppressor	radiosensitivity	[127]

In the study conducted by Yang et al., they demonstrated that the administration of anthracyclines or paclitaxel as chemotherapeutic treatments can stimulate exosome secretion by tumor cells, thereby abolishing chemosensitivity and promoting BCSCs’ expansion through the modulation of WNT/β-catenin and Notch signaling pathways and the activation of the transcription factor STAT3, leading to increased levels of miRNAs 378a-3p and 378d [114,128]. In this work, it was observed that after the administration of the first cycle of chemotherapy, STAT3 stimulation is induced, causing an increase in the levels of miRNA-378a-3p and miRNA-378d in the cytoplasm of BCSCs. These BCSCs release exosomes with a high concentration of both miRNAs, which travel through the bloodstream to the surviving cells. These cells uptake the exosomes and become insensitive to these drugs [114]. Their findings suggest that the chemotherapy-induced elevation of miRNAs 378a-3p and 378d is closely related to the patient’s treatment response and BCSC expansion. Furthermore, they show that chemoresistance can be reversed by reducing the levels of miRNAs 378a-3p and 378d through the inhibition of the enhancer of zeste homolog 2 (EZH2) protein, a stemness factor that drives oncogenic dedifferentiation and binds to STAT3 to activate it [114].

Another miRNA that may contribute to chemoresistance is the oncogenic role of exosomal miRNA-205 derived from chemoresistant BCSCs [121]. As proposed by previous studies, miRNA-205 is involved in numerous physiological and pathological processes related to cancer, such as cell proliferation [129], angiogenesis [130], EMT [131], and response to oxidative stress [132], making it essential for metastasis, cell self-renewal, and chemoresistance [133]. Zhao and colleagues found that elevated levels of miRNA-205 promote BCSCs’ proliferation, migration, and invasion by dysregulating the Akt pathway and tumorogenesis [121]. They demonstrated that tamoxifen-sensitive cells, when cultured in the presence of miRNA-205, internalized it and became drug-resistant. Moreover, researchers identified the target of this miRNA, suggesting that it targets the E2F Transcription Factor 1 (E2F1) gene by direct binding. This E2F1 gene encodes a transcriptional protein of the same name that modulates the tumor suppressor gene p53130. These findings suggest that by increasing E2F1 expression, all the oncogenic processes and chemoresistance associated with miRNA-205 can be reversed [121].

There are also underexpressed miRNAs in patients with BC, as occurs with miRNA-708-3p, a miRNA associated with chemosensitivity described by Lee et al. [117]. In this study, the researchers observed that BC patients have decreased levels of miRNA-708-3p compared to controls. The reduced expression of this miRNA was significantly associated with BC metastasis and chemoresistance [117]. In one of the conducted experiments, the researchers observed how the ectopic expression of miRNA-708-3p significantly inhibited metastasis and enhanced chemotherapy sensitivity. The authors also investigated the relationship between miRNA-708-3p and EMT in BC and were able to show that increased expression levels of miRNA-708-3p negatively regulate EMT activators, including ZEB1, CDH2, and vimentin [117].

The work of Hou et al. highlights the relationship between miRNA-1207, the Leucine Zipper Tumor Suppressor 1 (LZTS1) gene, and Taxol resistance in TNBC [122]. Previous studies have shown that miRNA-1207 is overexpressed in young BC patients and is positively correlated with the invasion, proliferation, and maintenance of stem cell properties of BCSCs [122,134]. Researchers found that miRNA-1207 was overexpressed in breast tumor tissue compared to control samples. Through various experiments using miRNA-1207 antagonistic molecules, they observed that LZTS1 mRNA expression levels increased due to the reduction in miRNA-1207, leading to the conclusion that LZTS1 is a target of this miRNA. They found that when LZTS1 expression levels decreased due to miRNA-1207 overexpression, taxol resistance emerged, which is associated with a poor disease prognosis. This is because LZTS1 regulates cell growth and apoptosis through the Akt/mTOR and PI3K/Akt signaling pathways. Therefore, a decrease in LZTS1 expression levels resulting from miRNA-1207 overexpression would confer taxol resistance [122].

miRNA-137 is another example of a dysregulated miRNA that affects resistance to conventional chemotherapeutic treatments, as shown in the work of Cheng et al. [123]. miRNA-137 acts as a negative regulator of oncogenesis reduces the expression of estrogen receptors in BC and decreases the proliferation and migration of BCSCs and breast tumor cells [135]. Cheng and colleagues identified that miRNA-137 reduces the expression of the glycoprotein follistatin-like 1 (FSTL1), a glycoprotein that was overexpressed compared to samples from other BC subtypes and control samples [123]. The results of this initial analysis led them to hypothesize that the overexpression of the FSTL1 protein might be responsible for chemotherapy resistance in these patients. The findings observed in this study allowed for the researchers to establish that dysregulations in the miRNA-137/FSTL1/integrin β3/Wnt/β-catenin signaling axis in BC are involved in the regulation of various processes related to the proliferation, differentiation, and migration of BCSCs, as well as chemotherapy resistance in the case of TNBC [123].

If we focus on the miRNAs that are dysregulated and involved in BCSCs in processes of radioresistance or radiosensitivity, it has been observed in a study by Troschel et al. that miRNA-142-3p expression levels decrease in BCSCs, leading to the upregulation of CSCs’ characteristics and radioresistance. This is because miRNA-142-3p, when expressed at normal levels, regulates the expression of Bod1, BRCA1, and BRCA2, all of which are involved in DNA repair when it is damaged by radiation. However, the dysregulation of miRNA-142-3p expression leads to the dysregulation of the mentioned genes, resulting in radioresistance [124]. Also, another miRNA related to the process of radioresistance is miRNA-29b-3p, which is involved in controlling the migration of BCSCs. This miRNA acts as a tumor suppressor gene by targeting DNMT3B, Bcl-2, PI3KR1, and AKT2, which radiosensitize the tumor by regulating DNA damage. Low expression levels of miRNA-29b-3p prevent these genes from being expressed correctly, leading to an increase in radioresistance in the tumor because the mechanisms regulated by these genes cannot be activated [125]. In the study conducted by Seok et al., it was demonstrated that miRNA-5088-5p expression levels increase in BC, leading to increased tumor malignancy, to reduce radiation-induced EMT, stemness, and metastasis by downregulating the Slug and DBC2. When miRNA-5088-5p levels increase, the expression levels of the Slug and DBC2 genes decrease, resulting in radioresistance in BC [126]. Finally, Sun et al. investigated the role of miRNA let-7d in radiosensitivity in TNBC. They observed that restoring let-7d expression levels inhibited BCSCs’ proliferation and self-renewal, as well as the activity of key genes related to cell proliferation and the Wnt/β-catenin pathway. Furthermore, let-7d increases TNBC sensitivity to radiotherapy, suggesting that restoring expression levels of the let-7 miRNA family, especially let-7d, may be a promising therapeutic strategy for TNBC patients [127].

## 6. Clinical Trials Related to miRNAs and BCSCs

Currently, in addition to all the research efforts aimed at identifying newly dysregulated miRNAs involved in breast cancer that can be used as biomarkers, whether for diagnosis, prognosis, or related to chemoresistance, clinical trials are being conducted to translate the results obtained from laboratory studies into clinical practice. Table 5 shows the most recent clinical trials analyzed in this review that involved miRNAs related to or described with BCSCs.

Numerous studies have shown that miRNA-155 is overexpressed in BC and is also correlated with a poor prognosis of the disease. Increased levels of miRNA-155 are associated with advanced stages of the disease [138], larger tumor size [139], lymph node metastasis, and invasiveness of tumor cells [140]. All this positions miRNA-155 as a potential biomarker for both diagnosis and prognosis. To translate these findings into clinical practice, in a real-world scenario, researchers Mojahed et al. conducted a clinical trial, the results of which have already been published [136]. The selected population consisted of women diagnosed with BC by imaging techniques and histopathology, within an age range from 20 to 60 years. For the control group, women between the ages of 20 and 60 had no lifetime history of cancer and no first-degree relatives. In addition, among the exclusion criteria for control cases, candidates were required not to have polycystic ovary syndrome. The clinical trial included all subtypes of BC with stages ranging from I to II. The study material used consisted of blood samples from both patients and control cases, from which serum was obtained and miRNA-155 was purified [136]. The results obtained in this study highlighted that, as affirmed by laboratory studies, miRNA-155 is overexpressed in patient samples compared to control cases. This overexpression was even higher when patients had lymph node metastasis. Furthermore, it was observed that there is no relationship between miRNA-155 expression and the positivity of hormone receptors, both PR, and ER, nor with HER2. Furthermore, no relationship was observed between miRNA-155 overexpression and the age of the patients. This clinical trial innovatively investigated the relationship between contraceptive use and miRNA-155 expression in patients, concluding that women who had never taken contraceptives had higher levels of this miRNA compared to those who had used them. The sensitivity and specificity of miRNA-155 as a biomarker obtained in this clinical trial were 77.78% and 88.89%, respectively [136]. The results of this study indicate that miRNA-155 expression can assist in the diagnosis and prognosis of BC, providing information about the likelihood of patients developing metastasis, with BCSCs being responsible for this [136].

In the clinical trial conducted by Anas and Hassan (NCT04720508), the expression of miRNA-373 and miRNA-425-5p is being evaluated in the serum of BC patients. Previous studies have reported that miRNA-425-5p is elevated in patients with this disease, indicating a poor prognosis for patients [91]. On the other hand, miRNA-373 could potentially serve as a biomarker for cellular invasiveness and lymph node metastasis, as low levels of this miRNA have been correlated with cell migration, disease recurrence, and BCSCs on multiple occasions [74,75]. In this study, Egyptian women aged 20 years or older diagnosed with BC have been selected as the experimental group, while the control group consists of healthy Egyptian women aged 20 years or older. Blood samples are collected from both groups, and the levels of the two studied miRNAs in serum are analyzed. This clinical trial is being conducted in two measurement phases. In the first phase, the levels of miRNA-373 and miRNA-425-5p are measured and compared between patients with BC and healthy control cases. In the second phase, correlations are sought between the expression of both miRNAs and clinicopathological characteristics of the patients, including stage, tumor grade, and positivity for hormone receptors and HER2. This clinical trial is currently ongoing, so results are not yet available.

In the clinical trial conducted by Salah and Sharawy (NCT05151224), the expression level of miRNA-21, that is related to BCSCs, as mentioned in this work, is being evaluated at different stages of disease treatment. This clinical trial includes women aged 18 years or older diagnosed with invasive BC of all subtypes, from whom blood samples are collected to assess miRNA-21 levels using RT-qPCR. The clinical trial consists of two 1-year-long stages. The first stage involves measuring and describing the expression levels of miRNA-21 before and after chemotherapy treatment, while the second stage focuses on measuring the relationship between miRNA-21 expression levels and clinicopathological characteristics of the tumor before and after treatment. This clinical trial is currently ongoing, so results are not yet available.

Another relevant clinical trial is the one conducted by Abdelghfour and Saleem (NCT04778202), in which they assess the potential of miRNAs 125-5p and 143-3p as non-invasive diagnostic biomarkers [31], as well as the relationship between the expression levels of these miRNAs and histopathological characteristics of the tumor, such as size, stage, molecular subtype, etc. Additionally, through this clinical trial, the researchers aim to improve the selection of treatment protocols, avoiding the resistance that arises in some cases due to the BCSCs population by making them more personalized for each patient based on the expression of these miRNAs. For this study, women aged 18 or older diagnosed with BC, who had not received radiotherapy, chemotherapy, hormonal therapy, or surgical treatment, were selected. For the control group, women aged 18 or older without a history of any type of cancer were chosen. Blood samples were collected and images were taken using magnetic resonance imaging to obtain samples. This clinical trial was conducted in two parts. In the first part, the expression levels of miRNAs 125-5p and 143-3p [31] were analyzed in both control and patient samples, and the results were compared to evaluate the diagnostic and prognostic potential of both miRNAs. In the second part, the expression levels of both miRNAs were compared with the imaging results to correlate the expression of these miRNAs with the clinicopathological status of the tumor, thus establishing better treatment protocols for each patient. Both stages have a duration of from 18 to 24 months. This clinical trial is currently ongoing, so results are not yet available.

In the study conducted by Müller et al., which is part of the clinical trial “Changes in serum levels of miR-21, miR-210, and miR-373 in HER2-positive breast cancer patients undergoing neoadjuvant therapy: a translational research project within the Geparquinto trial” (ID: NCT00567554), the dysregulation of miRNAs 21, 210, and 373 was evaluated before and after HER2 + BC patients received chemotherapy based on trastuzumab and lapatinib. As mentioned earlier in this work, the dysregulation of miRNA-21 is implicated in resistance to conventional therapies, as well as worse disease prognosis. On the other hand, elevated levels of miRNAs 210 and 373 are associated with an advanced clinical stage of the tumor, cell proliferation, migration, and invasion [137]. Furthermore, it was observed that after patients received the drugs trastuzumab and lapatinib, the levels of the three mentioned miRNAs increased, leading them to conclude that chemotherapy induces apoptotic cell death, increasing the concentration of circulating miRNAs, which could be an important indicator of sensitivity to these drugs [137].

## 7. Future Perspectives

CSC-targeted therapies hold great promise for improving the outlook for BC patients. These therapies aim to target and control BCSCs at their primary locations, thereby thwarting the recurrence instigated by metastatic BCSCs. miRNAs are small nucleic acids whose dysregulated expression, whether by overexpression or underexpression, can lead to oncogenic processes, cell invasion, and resistance to conventional treatments. These biomolecules are released by BCSCs into the bloodstream, either freely or within exosomes, and are characterized by their high stability, both in the tumor mass and in the blood, making their non-invasive acquisition simple and cost-effective. Hence, their application in clinical practice as potential biomarkers for this disease is of great interest. The positive relationship between BCSCs and miRNAs lies in the latter’s ability to maintain stem cells characteristics, increasing their self-renewal capacity, treatment resistance, and potential for differentiation into other cell types, incorporating miRNA-based therapeutics into CSCs-targeted approaches is expected to serve as a valuable strategy to inhibit tumor progression and metastasis.

Considering the crucial role of miRNAs in governing BCSCs, many studies have been conducted, often yielding contradictory results for the same miRNA. This lack of clarity in findings further complicates their clinical application. Nevertheless, the work done so far highlights that miRNAs exhibit greater precision and sensitivity than currently used biomarkers. Moreover, both parameters increase when, instead of studying individual miRNAs, panels consisting of a group of miRNAs are analyzed, establishing a miRNA signature for each subtype of BC. Despite the advances made in recent years, it is necessary to obtain more information about the causes of miRNA dysregulation, their mechanisms of action, their target genes, and how all of this influences the characteristics of BCSCs and tumor development. A better understanding of the functioning of these molecules would help to improve BC diagnosis and prognosis, allowing for more personalized treatments and reducing resistance to them. One way to obtain further realistic information about the functioning of these molecules is through clinical trials. Some clinical trials have been initiated in various regions of the world to analyze the relationship between specific miRNAs and BCSCs, as well as their utility in diagnosing and prognosticating BC patients. Most of these clinical trials have not yet concluded, so the available data are limited. Nevertheless, they will greatly expand the information available thus far.

To assist researchers in the search for new miRNA biomarkers of BC, as well as to store data from existing studies, multiple databases are currently being developed that compile all the information on miRNA sequences studied to date. The most important database regarding microRNAs is called miRbase [141], where one can download all the microRNA sequences, search for information about a specific one, or filter them based on the species. With the advent of Next-Generation Sequencing (NGS) technologies, there has been a significant increase in the submission of novel miRNA sequences to miRBase. Consequently, tools for researching miRNAs in the context of diseases are continually emerging. Novel miRNA biomarkers can be identified through manual curation, text mining (TM) of the literature, and predictions based on miRNA-target relations in expression data. There is an increasing development of database records and methods to forecast diagnostic and prognostic miRNA biomarkers for BC and BCSCs [142]. Other examples of these databases include mirnaQC, sRNAbench, and sRNAtoolbox. mirnaQC calculates absolute and relative values for various quality-related features of a set of miRNA sequences, enabling comparisons of miRNA sequences that facilitate the identification of low-quality samples, errors in data library preparation processes, and the validation of data quality to ensure their usability as controls [143]. sRNAbench and sRNA toolboxes are bioinformatics implements designed to accurately identify and quantify small miRNA sequences, enabling an understanding of how these molecules regulate gene expression in organisms and the biological functions in which miRNAs participate [144]. Continuing with the use of new tools or databases, a recent study has created the EVmiRNA database, which serves as a repository showcasing miRNA expression profiles within extracellular vesicles (EVs), including exosomes and microvesicles, that serve as vital mediators of cell-to-cell communication and hold promise as potential biomarkers in BCSCs [145]. Although several of the previous tools are well-maintained and have been integrated into new software solutions, it is foreseeable that future miRNA bioinformatics tools will focus on acquiring new knowledge related to miRNAs and the ability to effectively analyze high-throughput miRNA technology data [142].

## 8. Conclusions

miRNAs represent a highly interesting tool for diagnosing, prognosticating, and customizing treatments for BC, and more specifically to the BCSCs, to minimize treatment resistance as much as possible. Additionally, they can provide a wealth of information about the disease stage, tumor size, lymph node involvement, and the population of BCSCs within the tumor mass. Although a significant number of studies are currently underway, offering valuable data, they are not sufficient. This is because, at times, the findings from these studies are contradictory due to erroneous miRNA manipulation techniques and the lack of well-established protocols. All of this underscores the continued need for extensive research in this field and the development of new tools that enable researchers to identify the aforementioned errors in such databases. Although there is still work to be done, shortly, miRNAs could be applied in clinical practice to detect BC even before other current techniques, such as imaging techniques, do so. Additionally, they may help in personalized treatments for patients, as they provide valuable information about the development of the disease and the resistance to conventional treatments.

## Figures and Tables

**Table 1:** Targets and signaling pathways related to miRNA dysregulation in BCSCs.

BCSCs-Associated Pathway	MiRNA	MiRNA Acts as a Tumor Suppressor or Oncogenic miRNA	Target	References
Wnt/β-catenin	miRNA-600	tumor suppressor	SCD1	[21]
	miRNA-148a	tumor suppressor	WNT-1	[22]
	miRNA-140	tumor suppressor	SOX2/SOX9	[23]
	miRNA-3646	oncogenic	G3SK	[24]
	miRNA-142	oncogenic	APC	[25]
	miRNA-31	tumor suppressor	DKK1	[26]
	miRNA-29b	tumor suppressor	SPIN1	[27]
	miRNA-128-3p	tumor suppressor	NEK2	[28]
	miRNA-200c/141	tumor suppressor	HIPK1	[29]
	Let-7	tumor suppressor	ERα	[30]
	miRNA-125b	oncogenic	CK2-a	[31]
	miRNA-1	tumor suppressor	FZD7	[32]
JACK/STAT	miRNA-520c	tumor suppressor	STAT3	[33]
	miRNA-544	tumor suppressor	STAT3	[34]
	miRNA-93	tumor suppressor	STAT3	[35]
	miRNA-106a/b	tumor suppressor	STAT3	[36]
	miRNA-7	tumor suppressor	SETDB1	[37]
Notch	miRNA-34a	tumor suppressor	Notch1	[38]
	miRNA-130b-3p	tumor suppressor	DLL1	[39]
	miRNA-139-5p	tumor suppressor	Notch1	[40]
	miRNA-526b-3p	tumor suppressor	HIF-2*α*/Notch	[41]
	miRNA-200 family	tumor suppressor	JAG1/Maml2,3	[42]
PI3K/Akt	miRNA-221/222	oncogenic	PTEN	[43,44]
	miRNA-21	oncogenic	P85α	[45,46]
	miRNA-10b	oncogenic	PTEN	[47]
	miRNA-99a	tumor suppressor	mTOR	[48]
TGF-β	miRNA-181	oncogenic	ATM	[49]
	miRNA-128-2	tumor suppressor	SNAIL	[50]
NF-*κ*β	miRNA-181	tumor suppressor	PHLDA1	[51]
	miRNA-200b	tumor suppressor	IKK-B	[52]
	miRNA-448	tumor suppressor	NFKB	[53]

**Table 2:** Potential diagnostic miRNAs as biomarkers for BC.

MiRNA	MiRNA Acts as a Tumor Suppressor or Oncogenic miRNA	Target	References
miRNA-182	Oncogenic	BRCA1	[68,69,70,71]
miRNA-27a	Oncogenic	FOXO1	[72,73]
miRNA-373	Oncogenic	CD44, VEGF	[74,75]
miRNA-29b, miRNA-31	Oncogenic	PTEN	[26,76,77,78]
miRNA-21, miRNA-155	Oncogenic	various	[62,79,80,81,82]
miRNA-21, miRNA-155, miRNA-365	Oncogenic and tumor suppressor	various	[64]
miRNA-21, miRNA-155, miRNA-23a, miRNA-425-5p, miRNA-139-5p	Oncogenic and tumor suppressor	PTEN, FOXM1	[66,83,84,85,86,87,88]

**Table 3:** Potential prognostic miRNAs as biomarkers for BC.

MiRNA	MiRNA Acts as a Tumor Suppressor or Oncogenic miRNA	Target	References
miRNA-21-5p, miRNA-106b-5p	Oncogenic	GAB1, GNG12, HBP1, SESN1	[96,97,98,99,100,101,102,103,104]
miRNA-7641	Oncogenic		[105]
miRNA-10b	Oncogenic	E-cad	[89,106,107,108,109]
miRNA-126	Tumor suppressor		[110,111,112]
miRNA-21, miRNA-30c, miRNA-181a, miRNA-181c, miRNA-125b, miRNA-7, miRNA-200a, miRNA-135b, miRNA-22 and miRNA-200c	Oncogenic and tumor suppressor		[113]

**Table 5:** Clinical trials related to miRNAs involved with BCSCs.

Clinical Trial Title	Clinical Trial ID/References	miRNAs Studied	Clinical Trial Status
Clinical Evaluation of the Diagnostic Role of MicroRNA-155 in Breast Cancer	[136]	miRNA-155	Finished
Aberrant Expression of MicroRNA for Diagnosis of Breast Cancer	NCT04720508	miRNA-373, miRNA-425-5p	Not yet recruiting
Circulating microRNA 21 Expression Level Before and After Neoadjuvant Systemic Therapy in Breast Carcinoma	NCT05151224	miRNA-21	Not yet recruiting
Diagnostic and Prognostic Value of MicroRNA in Breast Cancer Patients	NCT04778202	miRNA-125-5p, miRNA-143-3p	Not yet recruiting
Changes in serum levels of miR-21, miR-210, and miR-373 in HER2-positive breast cancer patients undergoing neoadjuvant therapy: a translational research project within the Geparquinto trial	(NCT00567554) [137]	miRNA-21, miRNA-210, miRNA-373	finished

## Data Availability

Data sharing not applicable.
